# Comparison of the contents of selected elements and pesticides in honey bees with regard to their habitat

**DOI:** 10.1007/s11356-018-3612-8

**Published:** 2018-11-06

**Authors:** Monika Sadowska, Honorata Gogolewska, Nina Pawelec, Aleksandra Sentkowska, Beata Krasnodębska-Ostręga

**Affiliations:** 1 Faculty of Chemistry, University of Warsaw, Pasteura 1, 02-093 Warsaw, Poland; 2 Heavy Ion Laboratory, University of Warsaw, Pasteura 5A, 02-093 Warsaw, Poland

**Keywords:** Honey bees, Heavy metals, Xenobiotic elements, Pesticides

## Abstract

The inspiration for this study was the anxiety of Warsaw beekeepers, who raised the question whether location of hives in large urban agglomerations results in changes in concentrations of xenobiotics, toxic elements, and micronutrients in honey bees. Preliminary studies required elaboration of the research methodology, as the studied object is characterized by a low degree of homogeneity and the method of sample preparation affects obtained results. From many tested approaches, the use of washed and milled abdomens of the bees is recommended. Results obtained for such prepared samples are slightly lower than for whole bees, but their repeatability is higher, which enables easier interpretation of the trends and comparison of different locations. The contents of selected elements (As, Al, Cd, Co, Cr, Cu, Mn, Pb, and Zn) were compared in bees from urban and rural areas. The studies were supported by pesticides analysis. Also, it was checked whether these substances are accumulated on the surface or inside the bee’s body. The research indicates the markers of contamination: Al, As, and Cr on the surface and Cd inside the bodies of honey bees. The location of the hives does not influence significantly the content of “toxic,” nutrient metals and metalloids in bees (slightly higher levels of As, Al, Pb, and Cd were found in bees from urban areas). In terms of exposure to these elements and pesticides, the large city environment is not harmful for honey bees.

**Electronic supplementary material:**

The online version of this article (10.1007/s11356-018-3612-8) contains supplementary material, which is available to authorized users.

## Introduction

The threat of extinction of honey bees is an important subject not only in terms of ecology, but also for the economy. Usually, bees inhabit rural areas less often they are present in suburban regions. Regardless of the place of their existence, honey bees are exposed to a variety of factors that contribute to lowering of their immunity, which results in increased mortality of bees (Johnson 2008). Anthropogenic factors are of a great importance, as they contribute to accumulation of toxic elements in plants, contaminate pollen, nectar, and raw materials used for the production of bee products. Chemical protection of plants, which is an important aspect of crop production, also poses a threat for honey bees (García-Valcárcel et al. 2016). Especially dangerous is improper use of pesticides (Calatayud-Vernich et al. 2016), e.g., spraying of crops in bees’ feeding time. Plant protection products in relation to honey bees show toxicity by contact and ingestion (Porrini et al. 2003; Bargańska et al. 2016).

Nowadays, the presence of hives in large cities is quite common (http://pszczelarium.pl/; https://www.wien.info/en/sightseeing/green-vienna/urban-beekeepers; http://www.cityfarmer.info/category/bees/). A variety of plants are grown in towns, which prevents bees’ malnutrition, and less and different pesticides are used than on agricultural areas. However, the city is potentially a source of other kinds of threats, such as elements that are toxic at elevated levels (Pb, Mn, Zn, Cu, Co, Ni, Cd, As) (Porrini et al. 2003; Roman 2010; Zhelyazkova 2012; Lambert et al. 2012). Intoxication is most likely to occur when honey bees ingest contaminated pollen or nectar. However, xenobiotics transported on the body of the bee inside the hive can also be dangerous for the entire colony. Zinc accumulates mainly on the surface of the bees, which is explained by the presence of this element in dust depositing on bees’ hair (Leita et al. 1996; Zhelyazkova 2012). Exceeding of the optimum dose of Zn in the body of the insect leads to disruption of physiological processes, resulting in a negative impact on the functioning of the immune system. Additionally, nervous system disorders occur, manifested by difficulties with spatial orientation and recognizing of the hive or flowers (Buczek et al. 2008). In the case of cadmium, excess negative changes are observed in cellular defensive reactions, which results in a decrease of the number of phagocytes. Additionally, hemolymph proteins change in quantity and quality (Buczek et al. 2008; Zhelyazkova 2012). Cadmium accumulates both on the surface and inside the bee body. Higher cadmium contents were found in the external parts of the bees, which is associated with retention of Cd-contaminated dust and pollen on hairs (Leita et al. 1996). Lead is accumulated mainly inside the body in honey bees feeding in industrial areas (Leita et al. 1996; Lambert et al. 2012), while in natural areas, most of Pb was found on the surface of the body (Porrini et al. 2003). Lead affects honey bee’s immune system. Similar to Pb was the distribution of Cr: the less industrial area, the smaller the fraction of Cr found in honey bees’ tissues and the bigger the fraction of Cr accumulated on the surface of the body (Porrini et al. 2003).

Although Co, Mn, and Cu are micronutrients, the xenobiotic amount of these elements disrupts the defensive reactions of cells, reduces the phagocytic index, and changes the profile of low molecular weight proteins. Chemical compounds containing Cu, Mn, As, and Al are used in agriculture as pesticides (mainly insecticides). Pollinating insects are mainly exposed to pesticides when visiting melliferous plants. Major losses of honey bee colonies set near the crops treated with pesticides were reported by beekeepers (Stokstad 2012). The negative impact of pesticides on honey bees shows as physiological changes and genetic defects weakening the whole organism. Organophosphorus insecticides impair the neural transmission and muscle contraction. Bees lose their spatial orientation, and the ability to concentrate and learn (Decourtye and Devillers 2010; Eremia et al. 2010; Zhelyazkova 2012). Exposure to pesticides increases worker bee mortality causing disturbances in the functioning of the whole colony (Gill et al. 2012). With the increasing popularity of pesticide use, efforts have been made to protect bees from exposure to pesticides. First guidelines dealing with this aspect were published in the 1950s (Cluzeau 2002). Another point is that due to such exposure, honey bees can be used as bioindicators of pesticides residuals in the environment (Bargańska et al. 2014, 2016).

The driver for the study was the anxiety of the local manufacturers of honey. The beekeepers wanted to check the degree of contamination of honey bees existing in the city and compare it to areas urbanized in varying degrees. This knowledge would help to choose future locations of the hives and to recognize the dependencies between bees’ health condition and their contamination with some elements and pesticides. Therefore, the aim of this study was to propose a simple methodology of sample preparation, that will allow to assess the exposition of honey bees, and to apply it for comparison of the contents of selected elements in honey bees from hives located in urban and rural areas. Some metals were proposed by the partner institution Pszczelarium (well-known pollutants such as Zn, Cd, Pb, and Al), and some others (Cr, Cu, Co, As, and Mn) were added to the list of analyzed elements because they not only potentially affect the health of honey bees, but the accumulation of these elements in bees may depend on the place of feeding. Elemental analysis was supported by determination of pesticides. Also, it was checked whether these substances are accumulated on the surface or inside the bee’s body. Preliminary studies required elaboration of the research methodology, as the studied object is characterized by a low degree of homogeneity resulting from considerable environmental variation in metal and pesticides availability. Also, analyzing specific portions of the bees was investigated to determine whether the between-bee variation could be minimized. After choosing the most adequate method of sample preparation, honey bees from different locations were analyzed in order to determine whether location of hives in large urban agglomerations such as Warsaw (the city center, suburban area, small town near Warsaw) results in changes in concentrations of xenobiotics, toxic elements, and micronutrients (which are also toxic in higher doses) in honey bees. Among these analytes, we attempted to find a marker of contamination differentiating the honey bees from various areas.

## Materials and methods

### Samples

Honey bees were collected from hives located in areas that differ in degree of urbanization and industrialization. Urban bees were provided by “Pszczelarium,” which runs apiaries in a few places in Warsaw agglomeration. Bees used in this study originated from hives located in Pruszków (52° 10′ N 20° 47′ E), Galeria Mokotów (52° 10′ N 21° 00′ E), Białołęka (52° 20′ N 21° 03′ E), and Chełchy Iłowe (52° 58′ N 20° 57′ E). Pruszków is a small town close to Warsaw (part of Warsaw agglomeration) with ca. 60,000 inhabitants; Galeria Mokotów is a large shopping center in the city of Warsaw, surrounded mainly by office towers and apartment blocks; Białołęka is a suburban district of Warsaw, located on the right bank of the Vistula, with mainly family houses and many green areas; Chełchy Iłowe is a village in north Mazovia (90 km from Warsaw), in a rural/agriculture area, where mainly corn and wheat are grown. The samples were labeled as follows: Pruszków—urban area, Galeria Mokotów—city center, Białołęka—suburbs, Chełchy Iłowe—rural area. Collected honey bees were a crossbreed of Buckfast and Carniolan bees. Sampling was done once, at the same time in each location. At each location, few hundred bees were collected from few hives (bulk sample).

### Sample pretreatment before elements determination

**Fig. 1:**
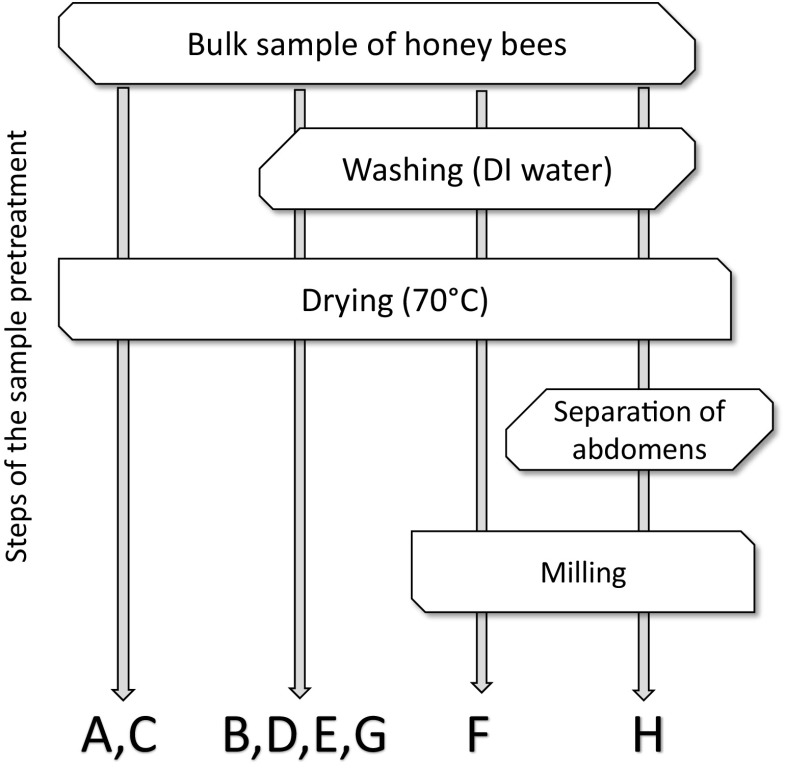
The scheme of sample pretreatment procedures used for preparation of honey bee samples for analysis

Bees used in this study were found dead at the bottom of the hives and transported immediately to the laboratory. To assess the impact of the method of sample preparation on the obtained results, the bees were prepared for analysis according to various procedures. The scheme of tested options of sample pretreatment steps is presented in Fig. 1. Part of the bees from each location were washed with deionized water (1 g of bees immersed in 30 mL of water for 5 min, then thoroughly rinsed). Then the bees were dried in 70 °C to obtain dry weight. Material taken for digestion consisted of whole bees or only abdomens, where most of the hemolymph is located. Hemolymph is a body fluid that transports nutrients and other substances, including investigated metals. The abdomens were obtained after manual removal of other parts of the body. From each bulk sample, the individual samples were taken for the analysis. The choice of samples was done based on the size of the bees. Five large (> 35 mg) and five small animals (< 35 mg) were selected for each sample, which was later subjected to digestion. Ten chosen bees were placed in a quartz crucible and 2.0 mL conc. nitric acid was added. The crucibles were put on a hotplate and heated to 100 °C. After the samples stopped foaming, the temperature was increased to 200–250 °C and the sample was evaporated to dryness. Then, 1.0 mL 30% H_2_O_2_ was added and evaporated to dryness again. The residue was dissolved in 5.00 mL of DI water and the solution was quantitatively transferred into a PP tube. For some analyses, the samples were prepared from bees grinded in an agate ball mill. In the case of milled bees, 300–330 mg of the material (ca. 10 bees) was put in a quartz crucible and the mineralization procedure was applied as described above.

The samples for the recovery study were prepared in a way intended to assure their repeatability: 750 mg of milled bees were put in a quartz crucible and 18.0 mL of conc. HNO_3_ was added. The crucible was heated until dissolution of the sample, and after cooling to room temperature, six portions of the solution (2.50 mL each) were pipetted into six crucibles (six samples of the same matrix composition and analytes content). Multi-element standard solution (50 μL containing 5.0 μg of each element) was added to three crucibles. All six solutions were heated again and evaporated to dryness (according to the digestion procedure), then 1.0 mL 30% H_2_O_2_ was added, evaporated to dryness again and the residue was dissolved in 5.00 mL of water.

All operations were done in a laminar flow box to avoid contamination, which is particularly important in the analysis of Al.

### Sample pretreatment before pesticides determination

The method described by Ghini et al. (Ghini et al. 2004) with some changes was adapted: 50.0 mg of ground bees were mixed with 5.00 mL of acetone and vigorously mixed for 10 min. The mixture was filtered through a Buchner funnel packed with layer of Celite 545 (Merck, Darmstadt, Germany) and washed with 10.0 mL acetone. Then 25.0 mL of coagulate solution (consisting of 1% ammonium chloride, 2% orthophosphoric acid (85%)) was added to the filtrate and left for 30 min. After filtration with Celite 545, the sample was diluted with 50.0 mL 2% aqueous sodium chloride (*w*/*v*) and extracted twice with 25.0 mL dichloromethane. For LC analysis, 1.00 mL of such extract was evaporated to dryness using gentle stream of nitrogen and dissolved in 1.00 ml of methanol. Then 5 μL of such prepared samples were directly injected to HPLC ESI MS/MS system.

### Determination of the total content of Al, As, Cd, Co, Cr, Cu, Mn, Pb, and Zn

Digested samples were diluted 10- and 100-fold with water and selected elements were determined using ICP MS. For spectrometric determination NEXION 300D mass spectrometer (Perkin Elmer, USA) with Meinhard-type nebulizer (400 μL min^−1^), cyclonic spray chamber and Ni cones were applied. Measurements were performed with the following parameters: sweeps, 3; readings, 1; replicates, 5; dwell time, 50 ms; ICP RF power, 1000 W, nebulizer gas flow, 0.88 L min^−1^; plasma gas flow, 13.1 L min^−1^; auxiliary gas flow, 1.40 L min^−1^; and monitored isotopes: ^27^Al, ^52^Cr, ^55^Mn, ^59^Co, ^63^Cu, ^66^Zn, ^75^As, ^111^Cd, ^206^Pb, ^207^Pb, and ^208^Pb. A quantitative analysis program was used to automatically correct the intensities of interfering isobaric and molecular ions. For quantitative determinations, the calibration curve method was applied. In case of Pb determination, the result was calculated as the mean value of the results obtained for each isotope. For each sample type (location, method of sample pretreatment), multiple digestions were performed (*n* = 4–6) and the final result was calculated as the mean value of results obtained for all *n* samples. Relative standard deviation (RSD) calculated for the final result was used as a measure of the inhomogeneity of the studied material.

### Determination of pesticides

Chromatographic analysis was performed using Shimadzu Liquid Chromatography system equipped with binary pumps LC-20 AD, degasser DGU-20A5, column oven CTO-20 AC, and autosampler SIL 20-AC connected to 8030 Triple Q mass spectrometer (MS). The system was controlled using the Lab Solution software. The pesticides separation was performed using Kinetex C18 column (100 × 2.1 mm, 2.6 mm) in 30 °C. Multi-linear gradient was used: 0–9 min 15% B, 10–13 min 50% B, 13–20 min 70% B, 29–40 min 90% B, where A is water and B is acetonitrile. The mobile-phase flow rate was 0.2 mL min^−1^ and the sample volume, which was injected to the column, was 5.00 μL. The MS system was equipped with the electrospray ionization source (ESI) operated in positive-ion mode. The ESI conditions were as follows: the capillary voltage 4.5 kV, temperature 400 °C, the source gas flow 3 L min^−1^, drying gas flow 10 L min^−1^. For each compound, the optimum conditions of multiple reaction mode (MRM) were determined. The method covered 30 pesticides. The optimal MS parameters for pesticides detected in the samples are shown in Table 1S. The analytes were identified by comparing retention time and *m/z* values obtained by MS and MS^2^ with the mass spectra. Each of the samples was analyzed in triplicate. Suitable values of log *P* were determined with the program Molinspiration from Molinspiration Cheminformatics (Sloven-sky Grob, Slovakia, available online at (http://www.molinspiration.com/cgi-bin/properties). Log *P* is a measure of hydrophilic character of the analyte: the more polar is the analyte the lower value of log *P* it has.

### Quality assurance

The elemental analysis was performed based on ICP MS determination after sample mineralization with nitric acid. The limit of quantification (LOQ) for the whole analytical procedure was calculated as (mean ± 10 SD) for six blank samples subjected to all stages of the methodology (mineralization, evaporation, and determination using ICP MS). The LOQs [μg g^−1^] were as follows: Al - 0.85; As - 0.014; Cd - 0.011; Co - 0.012; Cr - 0.048; Cu - 1.9; Mn - 0.11; Pb - 0.14; Zn - 4.1. For most of the studied elements, the blank values did not exceed 10–15% of the element’s content in the sample. The solutions after mineralization were properly diluted to assure that the concentration of the analytes is within the range of the calibration curves. The correctness and precision of ICP MS measurements were checked by analyzing certified reference material (surface water SPS-SW1 and SPS-SW2).

The reliability of digestion, which is the “weakest point” of the whole procedure, was tested using recovery study based on the honey bees matrix. Selected elements were determined in solutions obtained during mineralization of the sample with and without the addition of standard solutions. The solution obtained after dissolution of the sample was divided into six portions. Multi-element standard solution was added to three crucibles. The results of determination of selected elements in these solutions are presented in Table 1. The recovery of added standard of Co, Cr, Mn, and Pb was about 99%, and the recovery of Zn, Cd, Cu, As, and Al - about 105%.

**Table 1:** The recovery study. Standard solution containing 5.00 μg of each element was added to the bee sample during the digestion process. The results are presented as mean value [μg] (% RSD)

Solution after decomposition	Al	As	Cd	Co	Cr	Cu	Mn	Pb	Zn
Without standard	1.44 (1.7)	0.03 (7.6)	0.04 (8.1)	0.02 (4.2)	0.03 (4.2)	2.77 (5.0)	5.15 (3.5)	0.07 (6.6)	15.1 (5.0)
With standard	6.66 (3.5)	5.35 (3.1)	5.30 (3.4)	4.90 (2.8)	4.88 (3.1)	8.15 (5.6)	10.1 (3.4)	5.03 (2.7)	20.3 (4.8)
Recovery [%]	104	106	105	98	98	107	99	99	104

The calibration curve for pesticides determinations consisted of ten points (in triplicate) and it was established in the concentration range 1.0–20 ng g^−1^. The response obtained for the method was linear also for higher concentrations of the pesticides up to 100 times the LOQ. The correlation coefficients obtained for calibration curves for all pesticides were above 0.996. The limit of quantitation (LOQ) was evaluated as 10 times the baseline noise. The LOQs for the studied pesticides were in the range 4.5–5.1 ng g^−1^. The method was also tested in terms of accuracy and precision. Honey bee sample spiked at 1.5 μg g^−1^ was analyzed in triplicate. Recoveries were in the range 60–99% with RSD < 18%. The lowest recovery was obtained for alachlor (60%) and the highest for omethoate (99%).

Inter- and intra-day reproducibility of the instruments were also performed for the retention times and peak areas. The results were expressed as relative standard deviation (% RSD). The standard solution of all studied analytes was injected seven times daily for 1 week. The obtained intra-day RSD was 2.2% for retention times and 2.5% for the peak area. The inter-day RSD for the retention times of all peaks was < 3.5% and the coefficient variation for the peak area was < 4.3%.

## Results

The preliminary study revealed high inhomogeneity of the material especially in the case of Al determinations. In that study, one sample subjected to digestion and determination consisted of five bees, which appeared to be insufficient to be a representative sample. In further studies, to decrease the standard deviation, the number of bees taken for one sample was increased from 5 to 10. Additionally, the bees were subjectively selected according to their size to equalize the samples. In this study, one sample consisted of five large and five small bees. To confirm the results and check the repeatability of the procedure of sample preparation, two sets of five samples were prepared and the results were compared. Such study was done for urban (washed and unwashed) and rural (washed) bees (Table 2). The results obtained for these two sets did not differ statistically (*t* test), but the uncertainty of the results was relatively high. Bees are extremely heterogeneous research objects, because of the construction of their bodies and also individual characteristics (age, health status, time of emerging, feeding place). In case of such studies, it is important to assure the repeatability of the results and limit the influence of this inhomogeneity on obtained data.

Another aspect that is important for the reliability of the results is the step of sample preparation. Because mineralization of the bees is quite violent at the beginning of the process, wet digestion in an open system was used. The procedure consists of two steps carried until almost complete evaporation of the solution of oxidizing agent, and it is used for insect samples (Matin et al. 2016; Ghosh et al. 2016). Mineralization in an open system carries elevated risk of sample contamination or losses of the analytes caused by their volatility or deposition on the walls of the reaction vessel. For this reason, the recovery study was done to confirm the reliability of the decomposition procedure. The recovery study showed that the digestion procedure based on two steps of evaporation until dryness (in the presence of nitric acid and then hydrogen peroxide) is suitable for decomposition of bees and it assures obtaining correct results of ICP MS determinations (Table 1).

**Table 2:** Content of Al [μgg^-1^] in honey bees from urban and rural areas. Measurements were done for two independently prepared sets of samples (whole bees). Results are presented as mean value ± SD, *n* ≥ 4 (for each set of samples), and compared using *t* test

Sample	I set [μgg^-1^]	II set [μgg^-1^]	*t*_exp_	*t*_crit_ 95%	*t*_crit_ 99%
Urban, unwashed	8.6 ± 2.5	9.4 ± 1.1	0.5117	2.776	4.604
Urban, washed	7.5 ± 2.1	6.7 ± 2.7	0.3774		
Rural, washed	12.5 ± 6.5	10.4 ± 5.7	0.4099		

Selected elements were determined in bees collected from urban and rural areas (Table 3). Some of the bees were washed before mineralization to remove dust and other substances (e.g., pollen, honey) covering the surface of their bodies. Obtained results are characterized by high dispersion. The content of different elements varies greatly, and in some cases, it is so low (e.g., As in sample D) that RSD equal to 50% is acceptable. One-factor analysis of variance (ANOVA) was employed in the statistical evaluation to examine the differences between elements’ concentrations in bees prepared according to different schemes of sample pretreatment (Fig. 1) and from different locations. The ANOVA technique provides a convenient tool for establishing whether or not the data obtained via different sample preparation schemes belong to the same data population characterized by the normal distribution. The post hoc (Tukey–Kramer) tests coupled to the ANOVA mechanism allowed to indicate which data sets deviate significantly at 95% confidence level (Table 3). Results obtained for samples that were washed were lower or similar to results obtained for unwashed samples. However, washing of the samples rarely lowered the RSD of the results, showing that inhomogeneity is not a result of external contamination of the bees. Concentration of As and Cd was higher in bees from urban (unwashed) than from rural area. Co, Mn, Zn, and Cu, which are micronutrients, were found in similar quantities in urban and rural bees, regardless the washing procedure. The content of Al was a few times higher in rural than in urban samples, in the case of unwashed bees. Cr was accumulated mostly on the surface of rural bees.

**Table 3:** The content of selected elements in bees collected in different areas. Samples were prepared in two ways. The results are presented as mean value [μg g^−1^] (% RSD), *n* = 4–6. *A*, urban unwashed; *B*, urban washed, *C*, rural unwashed, *D*, rural washed

	A	B	C	D
Al	8.96 (19)^a^	7.08 (31)^a^	25.4 (19)^b^	11.4 (49)^b^
As	0.62 (35)^a^	0.21 (36)^b^	0.09 (33)^b^	0.02 (50)^b^
Cd	1.12 (23)^a^	1.10 (18)^a^	0.21 (19)^b^	0.16 (18) ^b^
Co	0.22 (25)^a^	0.18 (19)^ab^	0.20 (33)^ab^	0.13 (11)^b^
Cr	0.40 (13)^a^	0.26 (26)^a^	0.72 (16)^b^	0.22 (26)^a^
Cu	19.0 (23)^a^	21.3 (31)^a^	15.0 (27)^a^	14.9 (13)^a^
Mn	74.3 (30)^a^	74.4 (28)^a^	83.4 (23)^a^	85.0 (18)^a^
Pb	0.58 (38)^a^	*	0.50 (38)^a^	0.22 (37)^a^
Zn	136 (27)^a^	138 (21)^a^	139 (26)^a^	120 (10)^a^

*High dispersion of the results; ^a,b^mean values of element content (rows) denoted by the same letter did not differ significantly (*p* = 0.05)

Digestion of whole bees resulted in high dispersion of the results caused by obvious inhomogeneity of the sample. To increase the homogeneity two methods were applied: milling of the sample (whole bees) and milling after removing of the wings, legs, and head, and using only abdomens, which contain most of the vital organs and hemolymph. Hemolymph is insects’ body fluid transporting nutrient and other substances, including studied elements, to all parts of the body. Bees from two locations in Warsaw were analyzed and the results evaluated statistically with one-factor analysis of variance (ANOVA) coupled with the post hoc (Tukey–Kramer) tests (95% confidence level) (Table 4). Determined contents of most elements were comparable for whole bees and for the milled ones. Lowered results were obtained in case of Al and As in milled bees. In the case of As and Co, obtained results are close to the limits of detection of the applied procedure. However, milling significantly lowered the RSD of the results and despite some losses of the analytes during the milling process, for such prepared samples, it is easier to notice some trends in the obtained results. Using of abdomens only also lowers the RSD of the results (3–12% in comparison to 10–26% for whole bees). Comparison of the bees from two locations (Mokotów and Białołęka) showed comparable contents of Cd, Co, and Cr. The contents of Al, Pb, Zn, Mn, and As were higher in bees from Mokotów, and the content of Cu was noticeably higher in bees from Białołęka.

**Table 4:** Concentrations of selected elements in bees from two locations in Warsaw. Samples were prepared in various ways. The results are presented as mean value [μg g^−1^] (% RSD), *n* = (3–6)

Location	Sample form	Al	As	Cd	Co	Cr	Cu	Mn	Pb	Zn
Galeria Mokotów	Whole bees E	67.7^a^ (25)	0.34^a^ (16)	0.28^a^ (15)	0.33^a^ (10)	0.33^a^ (22)	9.64^a^ (26)	53.4^a^ (19)	0.64^a^ (19)	166^a^ (18)
	Milled F	29.8^b^ (3)	0.21^b^ (10)	0.24^a^ (9)	0.28^b^ (7)	0.28^a^ (9)	7.41^a^ (12)	52.7^a^ (10)	0.74^a^ (11)	137^ab^ (4)
Białołęka	Whole bees G	8.67^b^ (14)	0.10^c^ (25)	0.36^a^ (20)	0.23^b^ (14)	1*	20.0^b^ (17)	33.3^b^ (11)	0.23^b^ (23)	103^b^ (10)
	Abdomens H	11.0^b^ (5)	0.04^c^ (15)	0.94^b^ (8)	0.2*	0.16^b^ (12)	20.3^b^ (13)	43.5^ab^ (5)	0.33^b^ (14)	140^a^ (10)

*Approximate value; ^a,b,c^mean values of element content (columns) denoted by the same letter did not differ significantly (*p* = 0.05)

Except for elemental analysis, the qualitative and quantitative analysis of pesticides was performed to collect additional data regarding the condition of bees feeding in rural and urban regions. Firstly, the bees were washed with water to remove pollen and dust. The water used in this step of cleaning was collected and analyzed as well. The results obtained from HPLC MS/MS analysis confirmed the presence of pesticides both inside and outside the bodies of bees. The quantitative analysis of extracts of bees indicated that residue of pesticides was detected in all samples. In the case of rural bees, the concentration of oxamyl was three times higher in comparison to urban bees. In contrast, the concentration of azinophos-methyl was about 5 ng (in 1 g of the sample) higher in urban bees than in rural ones. Alachlor was detected in all studied samples at comparable concentration levels. It is not surprising, as alachlor is used both in agricultural areas (especially on corn and soybeans fields) and in the cities (as an herbicide). Small amounts of azinophos-ethyl and coumaphos were found only in urban bees. In contrast, thiamethoxam and methidathion were found only in rural bees. Water collected during washing of the bees samples contains pesticide residues different from those detected inside the bodies of bees. Only methidathion was detected both in the bees and in the water used for washing. In water, there were also detected: omethoate, qualiafos, imidacloprid, and oxydemeton-methyl. The comparison of results obtained for water and washed bees leads to some conclusions about the ability of migration of pesticides into the body of bees. The relationship between the polarity and the ability to penetrate deep into the body bees was investigated. It turned out that more hydrophobic pesticides were detected inside the bees (log *P* from to 4.84 for coumaphos and 3.67 for alachlor to 1.23 for thiamethoxam). Pesticides found in water samples were characterized by lower values of log *P* (from 0.84 for imidacloprid to − 5.67 for qualiafos). These studies confirmed that more polar pesticides can be found on the surface of the body of honey bees and more hydrophobic ones can go deep into the body of an insect.

## Discussion

Data obtained in this study lead to conclusions about analytical procedure of honey bee sample preparation that will guarantee obtaining reliable data regarding environmental pollution. Statistic evaluation shows that the washing process should be applied. Although determined concentrations of the elements are lower for washed samples, the RSD of the results decreases and the environmental trends are better noticeable (Table 3). The milling process and sieving of unground parts decrease the detected amount of trace elements but the RSD of the results noticeably decreases. Also, using only abdomens lead to decrease of the RSD values, and the losses of information on elements’ content were not so important. It is much easier to identify the trends when for the experiments washed and ground abdomens are used. On the basis of the results obtained for samples prepared in such way, conclusions can be drawn and discussed with already published data regarding some elements in the context of honey bees’ pollution versus feeding area.

**Fig. 2:**
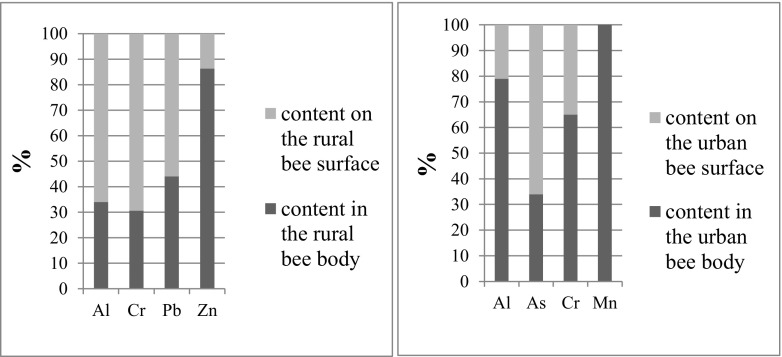
Distribution of Al, As, Cr, Pb, Mn, and Zn in rural and urban bees. The values are normalized to 100%

Bar graphs presented in Fig. 2 show the distribution of selected elements in bees obtained from rural and urban areas and show interesting trends. All data are shown in Table 3. The values of the elements’ content in unwashed bees were normalized to 100%, assuming that the content of the elements on the bee surface equals the content in unwashed bees minus the content in washed bees (the content in bee body). The content of the elements deposited on the bee surface is significant in case of Al (over 60%), Cr (30–60%), Co (about 30%), Pb (about 60%), and As (over 60%), for both rural and urban bees. For Zn, Cd, Cu, and Mn, the content on the bee surface is insignificant while compared to the content inside the body, regardless the feeding area. Low concentration of Cd on the surface of bee body is in agreement with literature data. Cadmium is mobile in soil, transported from soil to plants (Komarnicki 2005) and deposited directly in hemolymph (Zhelyazkova et al. 2004). It was reported that high content of Cd on the surface of bees may occur as a result of very occasional atmospheric contamination, especially when the samples are not collected in the vicinity of industrial areas (Perugini et al. 2011; Lambert et al. 2012). The study of Porrini et al. (Porrini et al. 2003) indicated similar trends for distribution of Pb and Cr, which are in higher concentrations on the surface than inside the body. It results from the fact that large part of Cr present in the environment is in the atmosphere (Seigneur and Constantinou 1995). A similar pattern was found in this study (Fig. 2, Table 3).

**Fig. 3:**
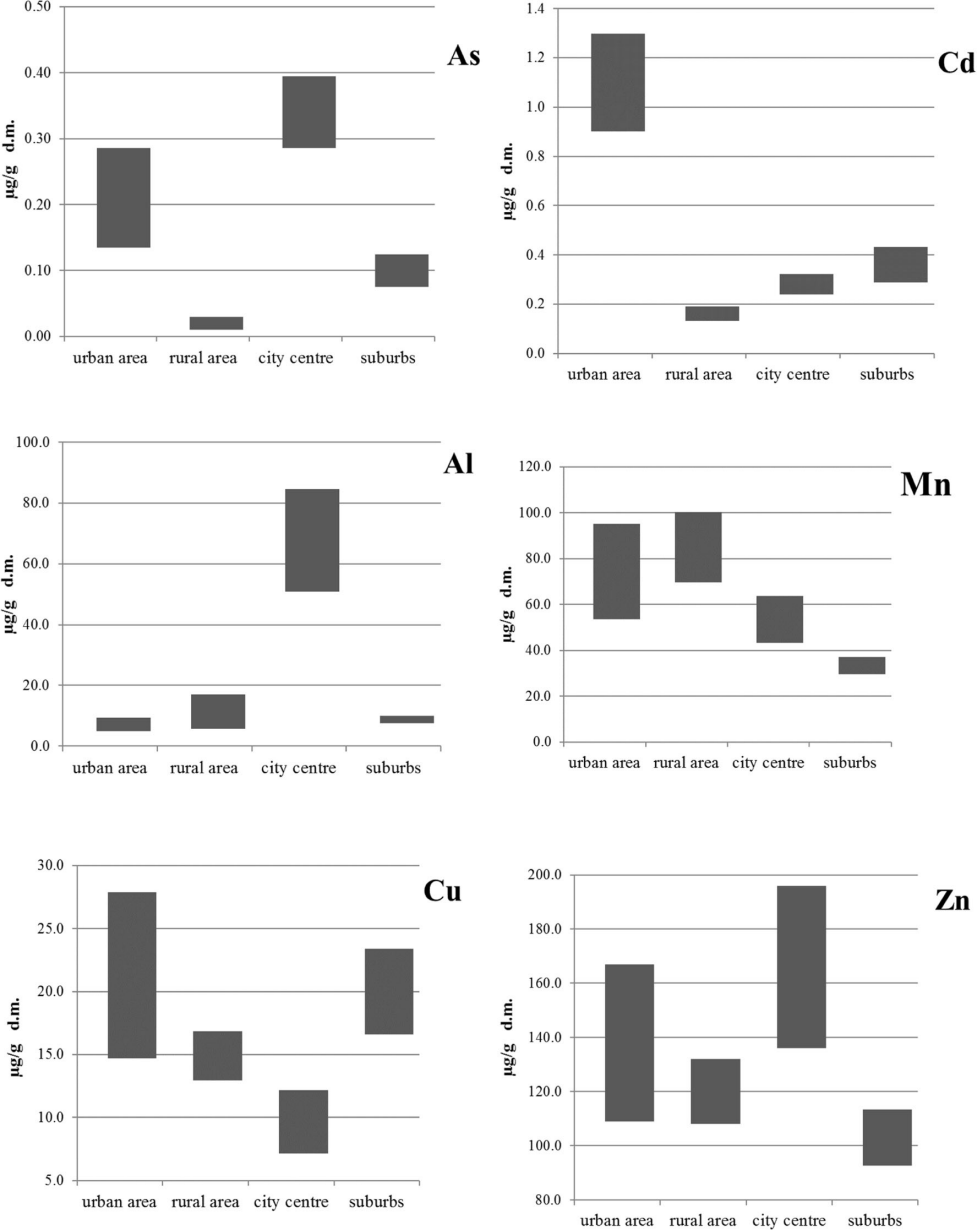
The contents of Cu, Cd, Zn, As, Al, and Mn in bees from various areas. Data are presented as a range appointed by the following values: mean value + SD and mean value - SD, *n* = 4–6

Figure 3 shows the ranges of content of selected elements in washed bee bodies collected in a small town close to Warsaw (urban area), in the vicinity of a village (rural area), in the center of Warsaw and in the suburban area of Warsaw. The contents vary depending on the feeding area of bees. The presented study as well as the literature data indicated that the content of Cu is approximately equal 20 μg g^−1^ (Fakhimzadeh and Lodenius 2000; Roman 2010), while in the case of Co, it is about 0.20 μg g^−1^ (Zhelyazkova 2012). Also, the Mn content is similar for all reported studies and it is about 50–70 μg g^−1^ (Zhelyazkova 2012). The contents of Pb and Zn in bees are slightly higher in samples collected in urban or industrial areas (Zhelyazkova 2012; Zarić et al. 2015) (Tables 3 and 4). The content of Pb detected during this study was below 1 μg g^−1^ for all samples, which is within the range already reported in the literature (0.52–1.25 μg g^−1^) (Conti and Botrè 2001; Roman 2010). Unexpectedly, the amount of Cd determined in washed bees collected in the small town—1.1 μg g^−1^ (Fig. 3) is much higher than in other localizations and also higher than Cd content reported by others for bees collected in industrial region (> 0.2 μg g^−1^) (Zhelyazkova 2012) and collected close to a large Polish town Wrocław (< 0.8 μg g^−1^). It was also higher than in bees collected in Polish province Warmia and Mazury, where the highest content of Cd was found in apiaries from central and northern part of the province (0.178 μg g^−1^ and 0.166 μg g^−1^, respectively), and the lowest in Piska Forest and at its perimeters (0.081 μg g^−1^) (Spodniewska 2007). But the content of Cd in rural bees (0.16 μg g^−1^, Mazovia village) was lower than in the sample collected from agricultural-woodland region in Poland - 0.36 μg g^−1^ (Roman 2010). The content of Cd found in urban bees is comparable with data presented in the study of Opole province (industrialized part of Poland) - about 1 μg g^−1^ (Roman 2010). The content of Cd in bees collected in Warsaw was slightly higher than in rural area but comparable to the content of Cd in bees collected in “clean” area (< 0.3 μg g^−1^) (Porrini et al. 2003).

In the study described by Zarić et al. (Zarić et al. 2015), there are presented data for unwashed samples. The bees were collected in highly urbanized area—Belgrade city, and in the rural region—agricultural/woodland region in Serbia. Therefore, the discussion of data obtained in this study in the context of the Serbia study is done based on unwashed samples. The content of Al in both Polish and Serbian bees collected in agriculture areas is similar (around 30 μg g^−1^) but in urban areas (capital cities), the content of Al is 10 times lower (9 μg g^−1^) in Warsaw. The content of Cd in bees from rural areas is similar (0.2 μg g^−1^) but it was relatively high in the town close to Warsaw, which is a region strongly influenced anthropogenically. The contents of Co (0.1–0.2 μg g^−1^) and Cu (15–20 μg g^−1^) in honey bees were practically equal in both regions. Chromium content was 2–3 times higher in Poland than in Serbia. Manganese concentration in the discussed areas was in the similar range (50–70 μg g^−1^). The concentration of Pb was slightly lower in Serbian samples, but in a similar range (< 0.8 μg g^−1^) to other published data (Conti and Botrè 2001; Roman 2010). In the case of Zn, in both rural and city areas, its concentration is similar, independently of the grade of urbanization, and the mean content is in the same range (90–150 μg g^−1^).

The results of pesticides analysis indicate that residue of different pesticides is present in all studied samples, regardless of the location. Therefore, honey bees can be very useful in the monitoring of environmental contamination (Porrini et al. 2003; Bargańska et al. 2016). But, more pesticides as well as higher concentration of them was found in rural bees samples. Unexpectedly, methidathion (organophosphorus pesticide prohibited in the European Union) was determined in concentration about 8 ng g^−1^ in rural bees. Bargańska et al. reported that this compound was present in 47.4% of their samples of honey bees collected in northern part of Poland (Bargańska et al. 2014). The reported concentration of methidathion was from above of LOD up to 22.4 ng g^−1^ (Bargańska et al. 2013, 2014). The analysis of washed bees and solution (water) obtained after washing the bees from pollen and dust traces, confirmed that more polar pesticides can be found on the surface of the body of honey bees and more hydrophobic ones can go deep into the body of an insect.

Pesticides that have been detected only in rural areas, such as thiamethoxam and methidathion, are characterized by very high hydrophobicity (values of log *P* 1.23 and 2.54 respectively). They penetrate the body of the insect, thus are more dangerous and may even cause death. The urban samples were dominated by slightly polar and polar pesticides, which did not penetrate into the body of the insect and which can be washed away with water.

## Conclusions

Summarizing, the method of sample preparation affects obtained results, and it is crucial that the samples are prepared the same way. Otherwise, the data cannot be compared. On the basis of this study, the use of washed and milled abdomens of the bees would be recommended for assessment of the level of exposure of the honey bees and comparison of pollution in various locations. Abdomen contains most of the hemolymph, washing is necessary for removal of external pollutants, and milling reduces the sample inhomogeneity. Results obtained for such prepared samples are slightly lower than for whole bees, but their repeatability is higher, which enables easier interpretation of the trends and comparison of different locations. To gain a possibility of comparison of the results with literature data, all samples were prepared for analysis according to a procedure similar to already described in the literature. It occurred that the contents of Cu, Zn, Mn, Pb, and Co are similar in honey bees feeding in all studied areas, both urban and rural. Nutrient elements in general should not be used as markers of pollution. Urbanization and industrialization of the environment result in higher levels of Pb and Cd in honey bees (on the surface). The differences, however, are not alarming. Significant part of Al, Cr, and As was found on the surface of bees’ bodies. The results support the hypothesis that location of the hives in a city does not influence greatly the content of “toxic,” nutrient metals and metalloids such as Al, As, Cd, Co Cr, Cu, Mn, Pb, and Zn in honey bees. However, Al, As, and Cr could be chosen as markers of contamination on bees’ surface and Cd content differentiates bees in terms of internal contamination. The analysis of pesticides shows that more polar pesticides can be found on the surface of the bodies of honey bees and more hydrophobic ones can go deep into the body. It confirms that the large city environment is more friendly for these beneficial insects.

## Electronic supplementary material

Table 1S Optimal MS/MS conditions for detected pesticides, their retention times, structure, and log *P* values. (DOCX 58 kb)
