# Supplementary material for: Comparison of the contents of selected elements and pesticides in honey bees with regard to their habitat
Source: Environ Sci Pollut Res Int. 2018 Nov 6;26(1):371–80. doi: 10.1007/s11356-018-3612-8 (PMC6318247; doi:10.1007/s11356-018-3612-8)
Supplement: Supplementary file 1 — Optimal MS/MS conditions for detected pesticides, their retention times, structure, and log P values. (DOCX 58 kb) [file 11356_2018_3612_MOESM1_ESM.docx]

**Table 1S** Optimal MS/MS conditions for detected pesticides, their retention times, structure and log*P* values.

| **Compund** | **Log P** | **Precursor**  **ion** | **Product**  **ion** | **Retention**  **time [min]** |
| --- | --- | --- | --- | --- |
| 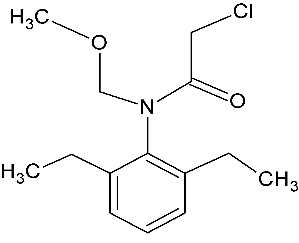  **Alachlor** | 3.67 | 270.1 | 238 | 17.0 |
| 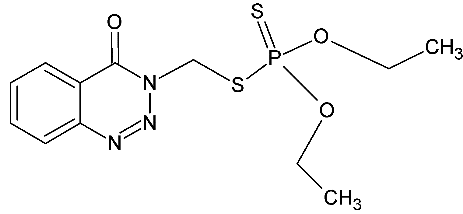  **Azinophos-ethyl** | 3.23 | 346.1 | 132 | 16.7 |
| 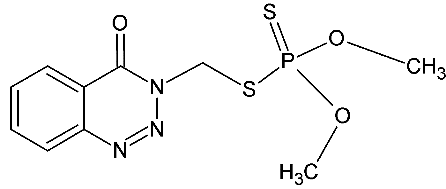  **Azinophos-methyl** | 2.48 | 318.0 | 132 | 14.8 |
| 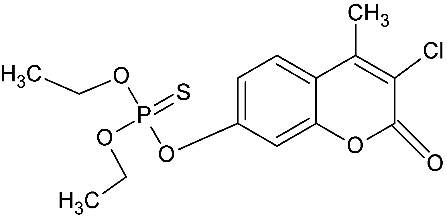  **Coumaphos** | 4.84 | 363.0 | 226.9 | 19.4 |
| 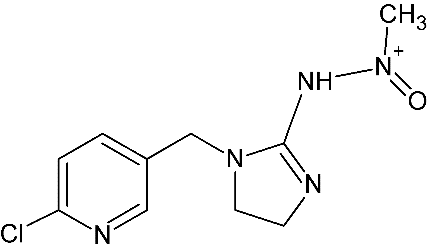  **Imidacloprid** | 0.84 | 256.1 | 209.0 | 5.70 |
| 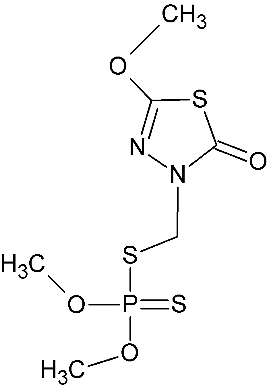  **Methidathion** | 1.79 | 303.0 | 145 | 14.4 |
| 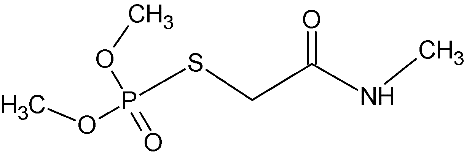  **Omethoate** | -0.79 | 214.0 | 124.9 | 2.35 |
| 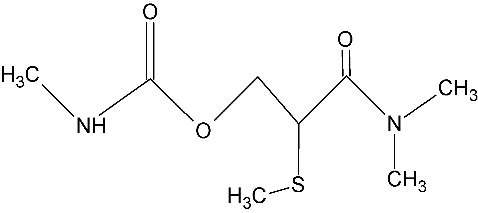  **Oxamyl** | -0.72 | 237.1 | 72.1 | 2.95 |
| 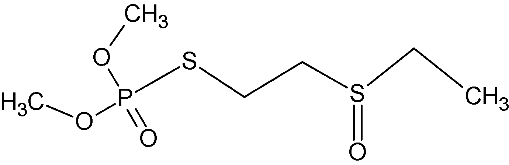  **Oxydemeton-methyl** | -0.52 | 247.0 | 168.9 | 3.45 |
| 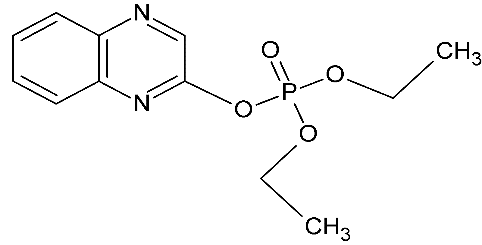  **Qualiafos** | -5.67 | 299.1 | 96.9 | 18.1 |
| 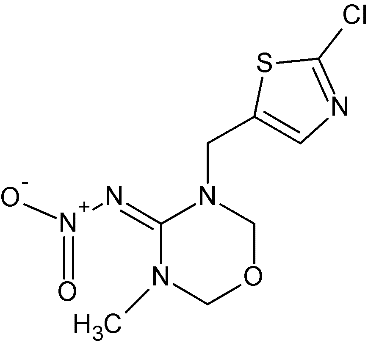  **Thiamethoxam** | 1.23 | 292.0 | 181.0 | 4.05 |
